# Tailoring the Meso-Structure of Gold Nanoparticles in Keratin-Based Activated Carbon Toward High-Performance Flexible Sensor

**DOI:** 10.1007/s40820-020-00459-5

**Published:** 2020-05-29

**Authors:** Aniruddha B. Patil, Zhaohui Meng, Ronghui Wu, Liyun Ma, Zijie Xu, Chenyang Shi, Wu Qiu, Qiang Liu, Yifan Zhang, Youhui Lin, Naibo Lin, Xiang Yang Liu

**Affiliations:** 1 Research Institute for Soft Matter and Biomimetics, Fujian Provincial Key Laboratory for Soft Functional Materials Research, Department of Physics, College of Materials, Xiamen University, Xiamen, 361005 People’s Republic of China; 2 Department of Chemistry, M. D. College, Parel, Mumbai, 400012 India; 3 Department of Physics, National University of Singapore, 2 Science Drive 3, Singapore, 117542 Singapore

**Keywords:** Wool keratin, Structure engineering, Metal nanoparticle carbon composite, Health monitoring, Flexible biosensor

## Abstract

Flexible biosensors with high accuracy and reliable operation in detecting pH and uric acid levels in body fluids are fabricated using well-engineered metal-doped porous carbon as electrode material. The gold nanoparticles@N-doped carbon in situ are prepared using wool keratin as both a novel carbon precursor and a stabilizer. The conducting electrode material is fabricated at 500 °C under customized parameters, which mimics A–B type (two different repeating units) polymeric material and displays excellent deprotonation performance (pH sensitivity). The obtained pH sensor exhibits high pH sensitivity of 57 mV/pH unit and insignificant relative standard deviation of 0.088%. Conversely, the composite carbon material with *sp*^2^ structure prepared at 700 °C is doped with nitrogen and gold nanoparticles, which exhibits good conductivity and electrocatalytic activity for uric acid oxidation. The uric acid sensor has linear response over a range of 1–150 µM and a limit of detection 0.1 µM. These results will provide new avenues where biological material will be the best start, which can be useful to target contradictory applications through molecular engineering at mesoscale.

**Electronic supplementary material:**

The online version of this article (10.1007/s40820-020-00459-5) contains supplementary material, which is available to authorized users.

## Highlights

- Materials with contradictory performance were prepared using wool keratin (WK).
- WK used for in situ preparation of AuNPs and N-doped carbon precursor as well.
- Two- and three-electrode flexible strip sensors designed for pH and UA detection.

**Electronic supplementary material:**

The online version of this article (10.1007/s40820-020-00459-5) contains supplementary material, which is available to authorized users.

## Graphical abstract

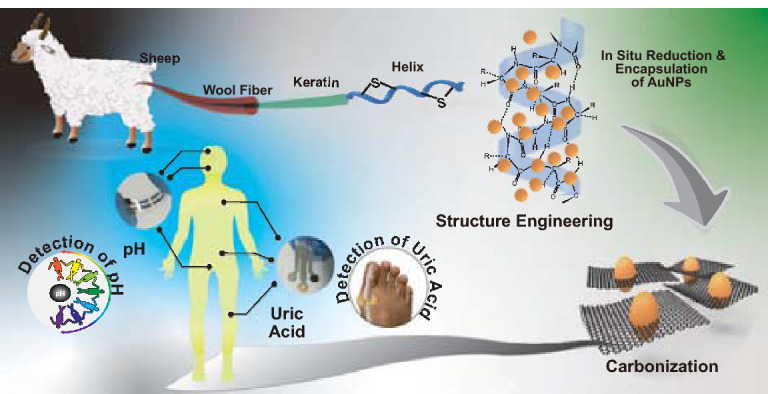

## Introduction

Biomarkers which are indicators for the health condition of an organism are generally sampled from body excretes such as sweat, saliva, urine, etc. [1]. The pH values of these fluids tell a lot about the health condition and is considered as important diagnostic information about kidney with type II diabetes, dermatitis, and fungal infections [2]. Another important biomarker, uric acid (UA) (2, 4, 6-trihydroxypurine) is a metabolite of purine derivatives excreted by kidneys [3]. The excess concentration of UA in the body has led to gout as well as cardiovascular and kidney diseases [3, 4]. Consequently, a quick and reliable detector is required to monitor pH and UA level. Interestingly, the detection of pH and uric acid is based on two contradictory principles, pH is relied on the measurement of redox potential, while uric acid detection is based on current response. Almost all solid-state pH sensors are developed by conducting polymers including polypyrrole, polyaniline (PANI), etc. PANI seems to be unique among these because of the configuration, which is of the A–B type, wherein B component is the basic heteroatom (N), eventually showing a high pH sensitivity by deprotonation of hydrogen ions [2]. However, PANI losses conductivity in neutral or basic solution. This issue does not allow it to be reused for several tests, and hence it is not a cost-effective sensor. Besides, UA estimation is depending on oxidation of UA and released e^−^ during oxidation. In this regard, materials like graphene, carbon nanotubes (CNTs), nitrogen-doped CNTs are used for the oxidation of uric acid with hosted metal nanoparticles due to *sp*^2^ hybridized carbon structure and zero bandgap. These materials, however, suffer from long and tedious synthetic procedures, fouling of catalyst, leaching of metals, etc.

Here, we engineered the molecular structure of wool keratin (WK) on mesoscale to manipulate properties as needed, for instance, pH and UA sensing materials with the high sensitivity, stability, and reproducibility. WK as a conductive carbon precursor and gold nanoparticles (AuNP) as an active metal component have been demonstrated. AuNPs are chosen as an active metal component, which not only accelerate the evolution of volatile components during carbonization resulting in highly porous carbon, but also enhance the total electric performance of the material. Under optimized conditions at 500 °C, conducting composite material, which mimics A–B type polymer, displays high pH sensitivity, whereas heteroatom-doped *sp*^2^-hybridized carbon composite material with zero bandgap has been fabricated at 700 °C carbonized condition under optimum parameters, which exhibits good conductivity and hence is excellent for UA detection.

## Experimental Section

Details about materials, preparation, and engineering of composite materials and characterization techniques are discussed in supporting information S1.

### Preparation of Two-Electrode and Three-Electrode Flexible Strip Sensor

#### Two-Electrode Silver Conducting Path

The two-electrode electrochemical sensor consists of the working electrode and reference electrode. In a typical fabrication protocol, the paper mask was prepared by laser cutting and attached on the PET film. The film is then subjected to magnetic sputtering to obtain a silver conducting pattern. Finally, the patterned electrode with silver conducting network was obtained by peeling of the paper mask. To prohibit cross-connections and to define the working area middle part of sensor covered by insulation film.

#### Three-Electrode Platinum Conducting Path

The three-electrode electrochemical sensor consists of the working electrode, reference electrode, and counter electrode. In a typical fabrication protocol, the paper mask was prepared by laser cutting and attached on the PET film. The film is then subjected to magnetic sputtering to obtain platinum conducting pattern. Platinum will act as a counter electrode during actual examination. Finally, the patterned electrode with platinum conducting network was obtained by peeling of the paper mask. To prohibit cross-connections and to define the working area middle part of sensor covered by insulation film.

#### Working Ink Preparation

The working ink solution is prepared for all carbonized composite materials i.e., AuNPs@NPWC-400, AuNPs@NPWC-500, AuNPs@NPWC-600, AuNPs@NPWC-700, AuNPs@NPWC-800. Accurately weighted carbonized composite material (5 mg) is dispersed in a 1 mL aqueous solution containing 10% nafoin binder. The solutions are treated for 10 min of ultrasonication to obtain a homogeneous dispersion.

#### Two-Electrode Sensor for pH Estimation

The working electrode was fabricated by drop casting method, wherein 10 mL of aqueous working ink was deposited on the working area and dried at 60 °C under vacuum for 1 h. The solid Ag/AgCl reference electrode was prepared by drop casting of 20 µL (10 mg mL^−1^) AgNWs aqueous solution on reference electrode area and subsequent annealing was carried out at 80 °C for 15 min. The Ag/AgCl electrode was prepared by chlorination of AgNWs by 0.1 M FeCl_3_ for 15 s. 40 µL of poly(vinyl butyral) (PVB) stock solution and 125 mg of NaCl in 2.5 mL of methanol was drop casted on the Ag/AgCl electrode.

#### Three-Electrode Sensor for UA Estimation

The working electrode was fabricated by drop casting method, wherein 10 mL aqueous working ink were deposited on working area and dried at 60 °C under vacuum for 1 h. The solid Ag/AgCl reference electrode were prepared by drop casting of 20 µL (10 mg mL^−1^) AgNWs aqueous solution on reference electrode area and subsequent annealing was carried out at 80 °C for 15 min. The Ag/AgCl electrode was prepared by chlorination of AgNWs by 0.1 M FeCl_3_ for 15 s. The third electrode i.e., counter electrode is platinum.

## Results and Discussion

**Fig. 1:**
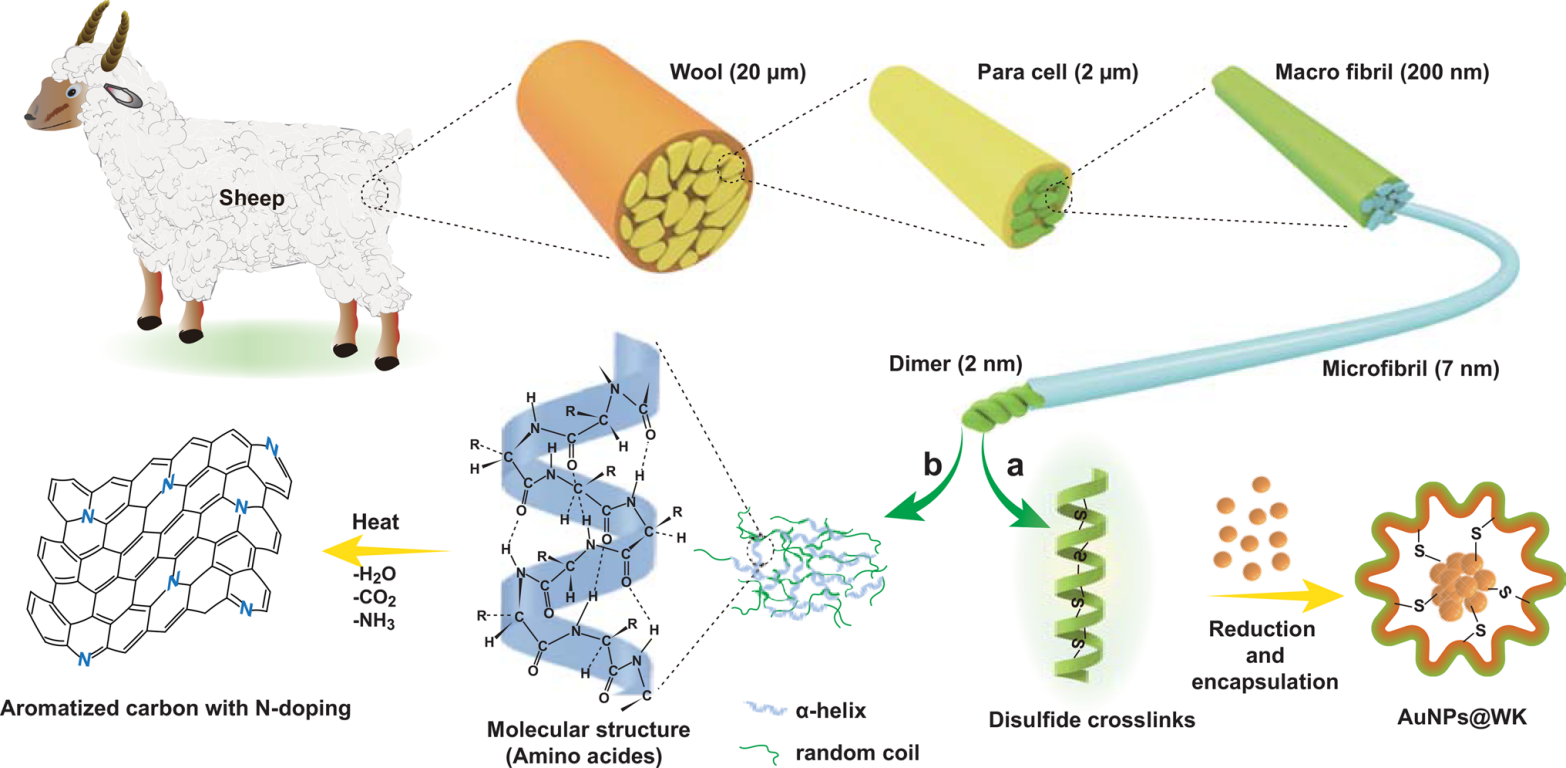
Schematic representation for the fabrication of **a** wool keratin-encapsulated gold nanoparticles and **b** nitrogen-doped aromatized carbon

**Fig. 2:**
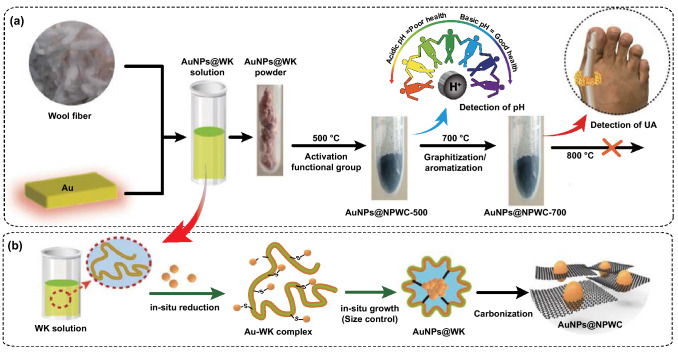
Preparation of well-engineered AuNP-doped porous carbonaceous materials. **a** Controlled preparation process of AuNPs@NPWC with structure manipulation to achieve diverse performance to target different applications. At 500 °C carbonization temperature, composite material possessing high % of N in the form of graphitic, pyridinic, and pyrrolic with carboxylic groups showing high sensitivity toward pH sensing, whereas at carbonization temperature 700 °C, composite material owed more aromatization, high porosity, tightly anchored, and well-dispersed spherical AuNPs, which is more sensitive for UA sensing application. **b** Schematic illustration showing in situ reduction and synthesis of AuNPs with subsequent encapsulation to the final carbonized AuNPs@NPWC composite material

The proposed in situ synthesis protocol of AuNP-doped mesoporous carbon composite using WK is outlined in Fig. 1. 5 mL aqueous solution of HAuCl_4_ (10 × 10^−3^ M) was added to 5 mL WK solution (2.5 wt%) under vigorous stirring, the pH of reaction mass is adjusted to 10–12, and retained at 45 °C for 12 h to form gold nanoparticles and wool keratin (AuNCs@WK) complex solution [5]. At the molecular level, WK has some significant aspects that can be beneficial for manipulation of the structure. In the first aspect, the WK molecule consists of up to 40% proteins (amino acid), which can transfer to the N-doped aromatic carbon through thermal treatment [6]. In another aspect, WK disulfide cross-linking, generally, it is important to stabilize the structure of wool fibers [7]. Some disulfide bonds get fragmented during the production of the WK solution. Each broken disulfide bond is divided into two mercapto groups (-SH), which are unstable and active and can be used as reducing agents [8]. Au ions can be reduced to atomic Au using these unstable mercapto groups and intercalated into layer space or absorbed on layer surface of regenerated WK, and tightly anchored into the unique precursor framework by strong interaction with amino groups (Fig. 2). Above prepared AuNPs@WK composite mixture is then exposed to freeze dry where water-based (or solvent based) suspensions transform to solid mass. As-prepared solid mass is then carbonized to different carbonization temperature between 400 and 800 °C under nitrogen atmosphere to achieve well-ordered porous carbon structure with improved electrical conductivity (Experimental section) [9]. The obtained AuNP-doped carbon composites are named AuNPs@NPWC-400, AuNPs@NPWC-500, AuNPs@NPWC-600, AuNPs@NPWC-700, and AuNPs@NPWC-800.

### Strategy of the Tailoring the Meso-structure

**Fig. 3:**
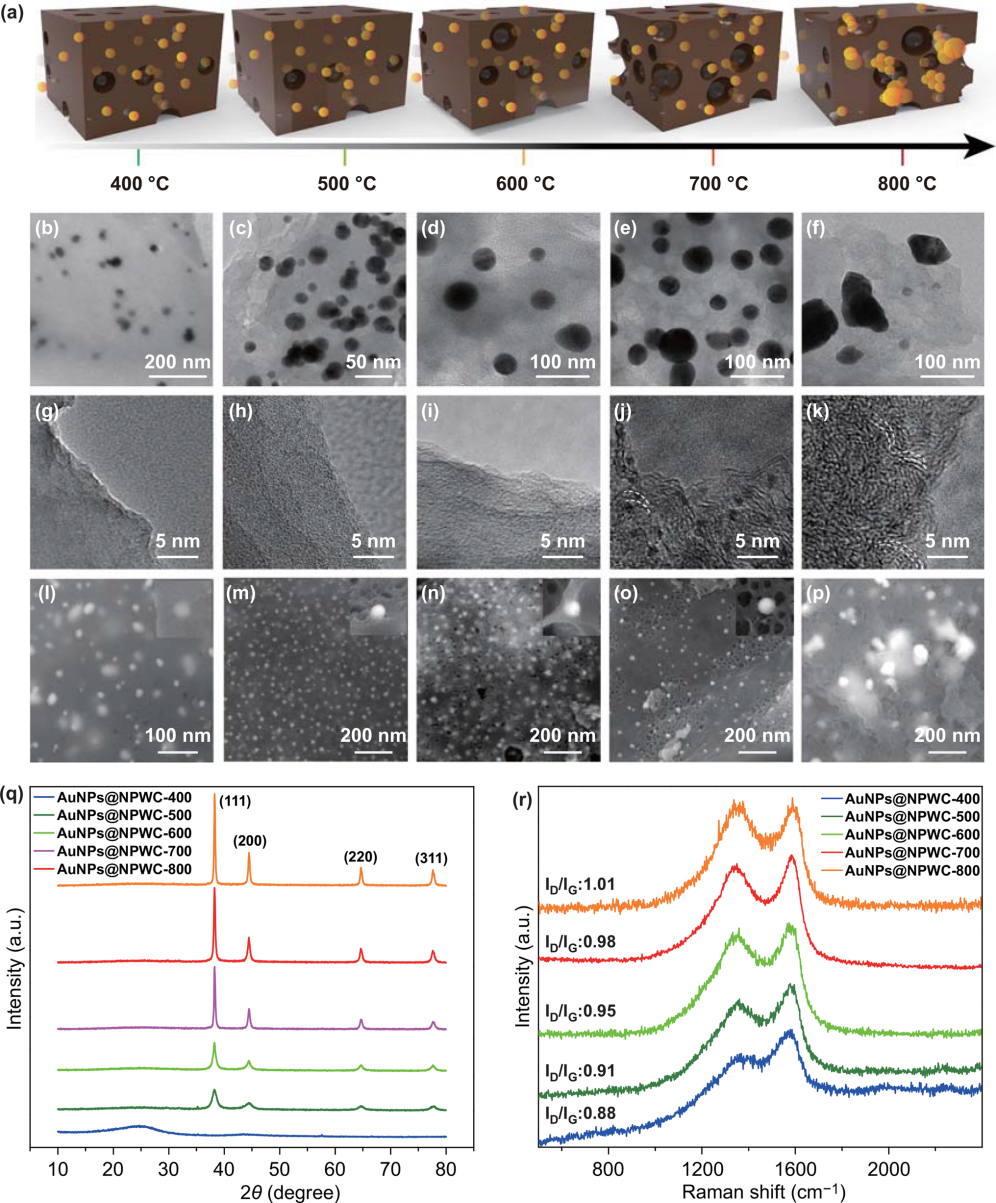
Effect of carbonization temperature from 400 to 800 °C on carbon skeleton and morphology of the AuNPs. **a** Schematic illustration. **b**–**f** TEM images showing effect of carbonization temperature morphology and dispersion of the AuNPs. **g**–**k** Effect on the aromatized carbon skeleton. **l**–**p** SEM images showing effect of carbonization temperature morphology and dispersion of the AuNPs. **q** Corresponding XRD pattern. **r** Corresponding Raman spectra

The degree of porosity is depending on the release of volatile matters [10]. The available AuNPs in the composite material catalyze and accelerate the evolution of CO_2_ and nitrogen in a basic form (amine) from the proteinases carbon precursor, resulting in high degree porosity and improved aromatization [11]. Figure 3a–e TEM images shows the morphology of AuNPs, in which spherical AuNPs with a size range of 20–40 nm were observed up to 700 °C carbonization temperature, which are well distributed and positioned in the nitrogen-doped porous carbon matrix, ensuring the maximum exposure of NPs, whereas agglomerated particles with arbitrary distribution were noticed at 800 °C. Figure 3f–j shows TEM images showing the effect of carbonization temperature on the carbon morphology, in which the percentages of porosity and lattice increase with temperature. The attached AuNPs were strongly anchored into the porous carbon matrix, which was confirmed by sonicating the composite sample for several hours in ethanol before TEM imaging. Figure 3k–o, SEM images of carbonized samples showed the increased degree of porosity in the composite materials with carbonization temperature from 400 to 700 °C that decline at 800 °C carbonization temperature. The declined porosity is due to the shrinkage of carbons at post-softening and inflammation temperatures, resulting in narrowing or closing pores [12]. The effect of temperature on the morphology of AuNPs was also examined; wherein in situ synthesized nanoparticles found well distributed in the matrix of porous carbon microstructure without any structural damage on morphology with increasing activation temperature from 400 to 700 °C. However, carbonization temperature above 700 °C resulted in disruptions of spherical morphology and agglomeration of AuNPs that leads to the deteriorated active surface area. The X-ray powder diffractometer (XRD) pattern (Fig. 3p) ratifies the crystallinity of the composite material. A diffraction peak at 2*θ* = 25.8° in the composite of AuNPs@NPWC exhibits the crystal graphite plane (002), which belongs to the hexagonal conjugated carbon structure. Furthermore, there are four strong diffraction peaks, which could be indexed to the (111), (200), (220), and (311) planes of Au nanoparticles. In comparison with the pattern of bulk Au from the Joint Committee on Powder Diffraction Standards (JCPDS No. 65-2870), the diffraction peaks from the synthesized Au nanoparticles are located in the same angles, confirming the formation and availability of Au nanoparticles in the composite. The *sp*^2^ domains structure and structure assembly of synthesized composite material have confirmed by the Raman spectra of carbonized AuNPs@NPWC (Fig. 3q). All the spectra showed two significant bands, the D band is usually attributed to the disordered carbon samples (e.g., defects on nanotube walls, vacancies, kinks, and heteroatoms); whereas, the G-band is attributed to C–C symmetric stretching [13]. Herein, the Raman spectra are characterized with the two bands obtained at 1360 and 1595 cm^−1^ could be ascribed to the D and G bands of AuNPs@NPWC nanocomposites. The obtained results reveal two significant observations, in one intensity of G-band increases with carbonization temperature that indicated increased in plane *sp*^2^–hybridized carbon. In the second observation, the increment in temperature from 400 to 700 °C showed the increase in *I*_D_/*I*_G_ ratio from 0.88 to 1.01 that fall down to 0.96 at 800 °C, these results are attributed to the degree of disordered surface that indicates the trend in porosity. Table S1 shows the elemental analysis of the composite materials prepared according to the varied carbonization conditions used in this study. The increase in carbon content and decrease in nitrogen and hydrogen contents is recorded with temperature increment. At carbonization temperatures higher than 700 °C, the little improvement in C/H ratio recorded, signifying no further changes in the chemical composition. Figure S1 and Table S2 show the BET apparent surface area, BET surface areas 27.09, 381.22, 452.65, 625.06, and 447.64 m^2^ g^−1^ are recorded for the products synthesized using furnace temperatures of 400, 500, 600, 700, and 800 °C, respectively. The steady rise in the degree of porosity and SBET values, which is correlated to the progression of compounds formed from the cross-linking reactions [14]. However, at high temperatures of 800 °C the opposite trend was noticed, which is demonstrated by the sintering effect of the volatiles and the shrinkage of the carbon structure [15]. These results regarding porosity, degree of aromatization and sintering of AuNPs are consistent with the TEM and SEM results observed previously. The effect of carbonization conditions on chemical state and the atomic conformation of composite materials were inspected by X-ray photoelectron spectroscopy (XPS) that exhibits four obvious characteristic peaks of C, N, O, and Au elements (Fig. S2). All carbonized composite materials were examined to understand the effect of carbonization conditions on the trend of structural evolution. N 1s spectra (Fig. S3) of the composite materials that deconvoluted with three types of nitrogen: pyridinic type N (398.6 eV), pyrrolic-type N (399.9 eV), and graphitic-type N (401.6 eV). Figure S4, C 1s spectra of the composite materials that deconvoluted with five types of carbon: C=C (284.5 eV), C–C (285.1 eV), C–O (286.3 eV), C–N (287.5 eV), and C=O (289.2 eV). Along with C and N content of O and Au were investigated (Figs. S5 and S6). Figure S5 shows O 1s spectras, in which the oxygen content consistently decreases with the increase in temperature, which is due to evolution of volatile content. Figure S6 shows the high-resolution XPS spectras of Au 4f, two distinct peaks at binding energies of 84.4 and 87.7 eV were observed, corresponding to Au 4f7/2 and 4f5/2, respectively. In contrast, four distinct spectra were noted in Fig. S6a, which may be due to Au(I) being linked to some thio groups at 400 °C carbonization temperature. Table S3 shows % elemental composition, the total carbon content increases with temperature, whereas total contents of N and O found decreased, which is attributed to the evolution of NH_3_ and CO_2_ groups with temperature. The existence of Au in the carbonized composite material was also confirmed by the XPS spectra of Au 4f (Fig. S2). Here, % Au composition found less at 400 °C carbonization temperature (NPs are buried in the composite matrix), which is almost similar in case of 500, 600, and 700 °C carbonization temperature. At 800 °C carbonization temperature, % Au composition is reduced drastically that is due to agglomerates formation which can be removed easily during washing treatment.

### A–B Type Polymeric Material for pH Sensor

**Fig. 4:**
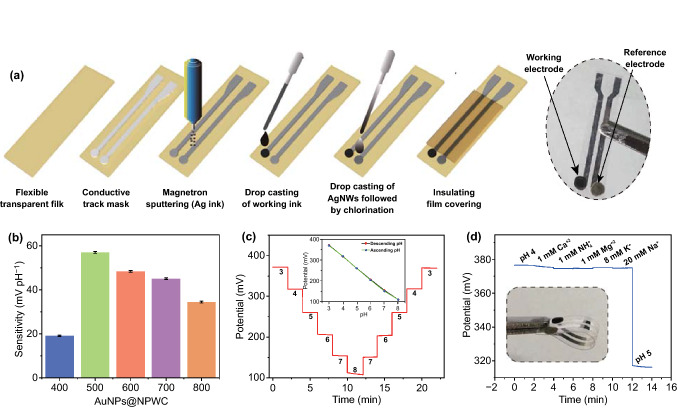
General strategy and response of pH sensor: **a** Fabrication of two electrode strip-based flexible sensor, **b** pH sensitivity of carbonized composite materials, **c** repeatability analysis and **d** selectivity of AuNPs@NPWC-500 modified pH sensor (inset showing hysteresis analysis)

The fabricated two-electrode flexible strip sensor can detect the pH of body fluids, which normally swings in between 3 and 8 (Fig. 4a). The five different pH sensor strips are prepared by changing working electrode material i.e., AuNPs@NPWC-400, AuNPs@NPWC-500, AuNPs@NPWC-600, AuNPs@NPWC-700, AuNPs@NPWC-800.

The nitrogen-doped carbonaceous composite material with COO^–^ functional group, possessing aromatized carbon skeleton (A-part) and N doping (B-part) reveals a unique π-conjugated structure, which acts similarly to A–B type polymeric material and mimics acid functionalized PANI [16], exhibiting high sensitivity for H^+^ ions (Fig. 4b). Herein, pH responses of as prepared sensors were investigated between ascending and descending range of McIlvaine’s buffer pH 4–6 (Figs. 4c and S7). Among all, AuNPs@NPWC-500 exhibits highest sensitivity of 57 mV/pH unit and 0.088% relative standard deviation (RSD). We found that the sensitivity of the composite material is dependent on the structure and chemistry. First, BET surface area (SBET), which is considerably good and much higher in AuNPs@NPWC-500 compared with AuNPs@NPWC-400 (Fig. S1). Next, the total content of pyridinic N1 and pyrrolic N2 in 500 °C carbonized material is the most, which is transferred to the stable graphitic N3 and oxidized N4 upon increasing the carbonization temperature (Table S1, S3, Fig. S3). The high content of pyridinic N1 and pyrrolic N2 nitrogen are more susceptible toward H^+^. Besides, the high content of C–O, and C=O is recorded in AuNPs@NPWC-500, which reduces in the form of CO_2_ with temperature (Fig. S4). The pH responses are also compared with the carbonized material without AuNPs i.e., NPWC-500, the sensor shows less sensitivity than that of the AuNPs@NPWC-500 (Fig. S8). In the absence of AuNPs material, carbonization process does not experience catalytic effect of AuNPs, hence, resulting in less porosity, eventually less SBET. In conclusion, the high deprotonation of H^+^ is strongly dependent on high content of pyridinic and pyrrolic nitrogen with extra carboxylic species and good porosity, AuNPs@NPWC-500 among all five is a better combination of optimum SBET and π-conjugated N-doped aromatic structures.

The further examination and applications of strip pH sensor are performed by using AuNPs@NPWC-500 as a working electrode material. Since pH in human body fluids normally swings in between 3 and 8 [17–19], the optimized AuNPs@NPWC-500 based pH sensor repeatedly examined for descending and ascending pH from 3 to 8, exhibits an average slope of 52.97 mV/pH unit (Fig. 4c). The sensor is highly selective to H^+^ ions with potential variation of 3.3% when 1 mM Ca^2+^, 1 mM NH^4+^, 1 mM Mg^2+^, 8 mM Mg^2+^, 8 mM K^+^, or 20 mM Na^+^ are added (Fig. 4d). For the investigation of reproducibility, four parallel sensors are fabricated, the absolute potentials range from 373.8 to 377.7 mV at pH 3 and sensitivities vary from 55.2 to 57 mV/pH unit of concentration (Fig. S9). These sensors show RSD of 0.88%, which is very less in comparison with the earlier reports (Table 1). The calculated sensitivity values were used to prepare a standard calibration curve in order to measure the pH of body fluids i.e., urine and sweat (Fig. S10). The examined stability of the pH sensor (Fig. S11) shows only 0.9 mV deviation over 8 h measurement, which is found better than earlier claims using PANI as a pH sensitive material, for instance Ali Javey and coworkers reported deviation of 0.7 mV h^−1^ and Park et al. reported 3.0 mV h^−1^ deviation [16, 20]. The main hurdle during the quantitative analysis of body fluids is the influence of Cl^−^ on solid-state Ag/AgCl reference electrode. This issue has overcome by PVB-based Ag/AgCl reference electrodes, which is found to be relatively stable because the PVB layer contains Cl^−^ and is resistant to alteration in Cl^−^ concentrations [16]. The above examination outcomes show that the present pH sensors offer a precise and reliable analysis competency.

**Table 1:** Comparison of different electrochemical sensor proposed for the determination of pH

pH Sensing material	Sensitivity (mV/pH)	RSD %	References
Graphene oxide	40	0.15%	[21]
Poly(terthiophene benzoic acid)/AuZn alloy oxide	58.5 ± 0.87	2.0	[22]
PAN/carbon fiber	50.1	3.72	[23]
PANI/gold	62.5	1.0	[16]
AuNPs@NPWC-500	57	0.088	This work

The practicability of the pH sensor was studied by examination of body fluids, wherein pH of human sweat and urine samples were examined and validated with a commercial pH meter (M/s. Thermo scientific). Quantitative estimation of body fluids using sensors is computed by use of a calibration curve obtained from potential values for buffer solutions. For real sample analysis, 5 sweat and 5 urine samples from volunteers were received. pH in sweat and urine measured by pH sensors shows maximum variation of 0.72% and 2.09% in sweat and urine from a commercial pH meter (Table S4). These differences are comparatively minor related to ordinary pH range of body fluid.

### Material with Zero Bandgap for UA Sensor

**Fig. 5:**
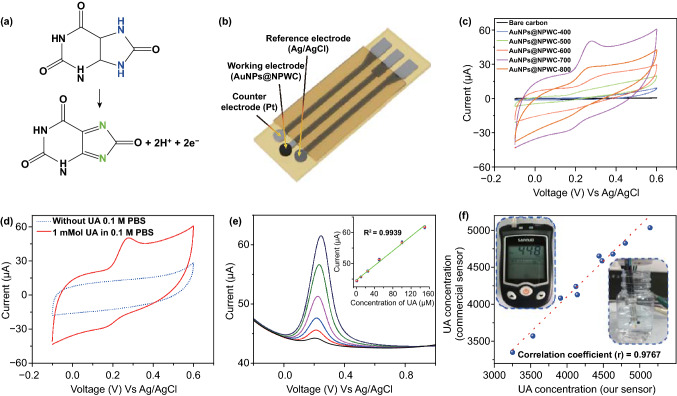
General strategy and response of UA sensor: **a** Reaction scheme showing non-enzymatic electrocatalytic UA oxidation reaction. **b** Schematic illustration of three-electrode flexible strip-based sensor. **c** Cyclic voltammograms of 1.0 mM UA in 0.1 M PBS (pH = 7) using different electrodes. **d** Cyclic voltammograms of 1.0 mM UA in 0.1 M PBS (pH = 7) and without UA only 0.1 M PBS (pH = 7) using AuNPs@NPWC-700 modified sensor. **e** DPV curves of AuNPs@NPWC-700 electrode in different concentrations of UA (1–150 µM) containing 0.1 M PBS (pH = 7) solution, the plot of oxidation currents versus the concentration of UA (inset). **f** Correlation between the developed biosensor and the commercial standard UA meter

In contrast to the pH sensor, we also target UA sensor, wherein the material is designed to show high electrocatalytic activity toward UA detection that would offer high sensitivity and selectivity. Principally, UA undergoes easy oxidation at electrodes modified by catalyst in aqueous solutions with release of e^−^ [24]. Our non-enzymatic electrocatalytic measurements showed that the as-prepared AuNPs@NPWC-carbonized material with hosted metal nanoparticles due to *sp*^2^ hybridized carbon structure and zero bandgap would be the best alternative non-enzymatic electrocatalytic material for electrocatalytic oxidation of UA (Fig. 5a).

The three-electrode flexible strip-based electrocatalytic system is designed to determine UA concentration in an aqueous medium which possesses a working electrode, Ag/AgCl reference electrode, and counter platinum electrode (Fig. 5b). Initially, five sensors were prepared with different working electrodes using carbon materials carbonized at different temperature (AuNPs@NPWC-400, AuNPs@NPWC-500, AuNPs@NPWC-600, AuNPs@NPWC-700, AuNPs@NPWC-800). Figure 5c presents a voltammograms obtained using different sensors in 0.1 mM UA containing phosphate buffer pH 7. As observed, the sensor using AuNPs@NPWC-700 composite exhibits a high peak current compared with those of other materials. In particular, AuNPs@NPWC-700 reveals extra porosity (SBET: 625.0586 m^2^ g^−1^ Table S2), increased aromatization (Fig. 3i, p, q) and well-dispersed AuNPs among all (2*d*,n) and hence exhibits excellent electrocatalytic performance toward UA oxidation. Although, AuNPs@NPWC-800 has more aromatized carbon structure, due to the reduced SBET area (447.6388 m^2^ g^−1^) and disruption and agglomeration of AuNPs (Fig. 3e, o), the corresponding sensor exhibits less response toward UA oxidation. From the deconvoluted XPS spectra of all the carbonized sample (Figs. S2 and S4), C1s spectra show increased C=C content with temperature, indicating high degree graphitization and eventually current response. The deconvoluted N1s spectra show increased percentage of graphitic nitrogen with temperature (Fig. S3), N doping in plane helps to anchor AuNPs and improves the current response as well. For the voltammograms obtained at AuNPs@NPWC-700 electrode with or without 1 mM UA (Fig. 5d), the significant anodic peak is recorded using AuNPs@NPWC-700 that attributed to the oxidation of UA. From these observations, we can conclude that AuNPs@NPWC-700 sensor has the strongest sensitivity toward electrocatalytic estimation of UA.

Differential pulse voltammetry (DPV) was carried out in the potential range of − 0.2 to 1 in PBS (pH 7.0), in which oxidation peak potential of UA is recorded at 0.22 V (Fig. 5e). The corresponding linear responses between peak current and UA concentration is detected from 1 to 150 µM with detection limit of 0.1 µM (*S*/*N* = 3). The sensitivity of the AuNPs@NPWC-modified sensor was calculated using the DPV results, which is 3.716 µA µM^−1^ cm^−2^. To the best of our knowledge, the presented linear detection range obtained using AuNPs@NPWC-700 is excellent compared with the earlier reported metal nanoparticles carbon-based electrodes of UA detection (Table S5). After 30th day, the AuNPs@NPWC-700 modified biosensor shows 90.15% oxidation peak current value to that of the first day indicating high stability and long-term durability (Fig. S12). This exceptional stability is attributed to the leaching free electrocatalytic material, which is confirmed by SEM (Fig. S13), in which no obvious change observed in the materials before and after use. An interference study was showed using DPV analysis in the voltage range of − 0.25 to 0.75 V against (Ag/AgCl). The possibly interfering species glucose (0.1 mM), ascorbic acid (0.1 mM), urea (0.1 mM), and lactic acid (0.1 mM) were selected. There is no obvious peak recorded for the other possibly interfering species except uric acid (Fig. S14), which indicates the selectivity of as prepared AuNPs@NPWC-700 electrode toward electrocatalytic oxidation of UA.

An efficiency and accuracy of the developed biosensor were performed for real sample estimation. The correlation curve between the UA concentrations in human urine samples obtained from the proposed strip-based sensor and the commercial UA detecting device is investigated (Fig. 5f), all the examinations were performed at room temperature and the biosensor response on the UA concentration (calibration curve) is presented. For real sample analysis, 10 urine samples from volunteers were received and diluted to 1:50 by PBS (pH = 7). The current response for each diluted sample was examined using DPV analysis, and the final concentration of UA is calculated by using calibration curve Fig. 5e (inset). The UA concentration examined by our sensor (axis X) and the commercial UA detector (axis Y) (Fig. 5f, Table S6) has a correlation coefficient of 0.9767. The demonstrated results revel the high reliability and accuracy that are highly acceptable.

## Conclusions

In summary, we present a simple, economic, and sustainable in situ synthesis method to prepare a novel AuNPs@NPWC composite material using WK as both a novel carbon precursor and a stabilizer. The targeted properties of composite material for a particular application were accomplished by structural engineering. A series of composite materials were prepared and screened for pH and UA sensing applications in the form of flexible sensors. AuNPs @ NPWC-500 reveals a high-N content in pyridinic and pyrrolic forms that mimic A–B type conductive polymeric structure, which displays best pH sensing performance. However, with extra porosity, well-dispersed AuNPs and increased aromatization, AuNPs@NPWC-700 exhibits easy and quick electron delocalization between the AuNPs and π-conjugated aromatized carbon skeleton, thus the corresponding sensor shows high sensitive toward UA sensing. The sensors allow high accuracy and reliability toward detection of pH and UA in body fluids including sweat and urine, which are significant with respect to the commercial devices. Our acquired results offer a new platform for in situ fabrication of metal substrate composite and structural engineering to manipulate properties for diverse electrocatalytic and catalytic applications.

## Electronic supplementary material

Below is the link to the electronic supplementary material.

Supplementary material 1 (PDF 2134 kb)
