# Supplementary material for: Tailoring the Meso-Structure of Gold Nanoparticles in Keratin-Based Activated Carbon Toward High-Performance Flexible Sensor
Source: Nanomicro Lett. 2020 May 29;12:117. doi: 10.1007/s40820-020-00459-5 (PMC7770875; doi:10.1007/s40820-020-00459-5)
Supplement: Supplementary file 1 — Supplementary material 1 (PDF 2134 kb) [file 40820_2020_459_MOESM1_ESM.pdf]

Supporting Information for

Tailoring the Meso-Structure of Gold Nanoparticles in Keratins-Based Activated Carbon toward High-Performance Flexible Sensor

Aniruddha B. Patil[1, 2,] *, Zhaohui Meng[1], Ronghui Wu[1], Liyun Ma[1], Zijie Xu[1], Chenyang Shi[1], Wu Qiu[1], Qiang Liu[1], Yifan Zhang[1], Youhui Lin[1], Naibo Lin[1,] *, Xiang Yang Liu[1,3,] *

[1]Research Institute for Soft Matter and Biomimetics, Fujian Provincial Key Laboratory for Soft Functional Materials Research, Department of Physics, College of Materials, Xiamen University, Xiamen 361005, People's Republic of China

[2]Department of Chemistry, M. D. College, Parel, Mumbai- 400012, India

[3]Department of Physics, National University of Singapore, 2 Science Drive 3, 117542 Singapore, Singapore

Aniruddha B. Patil and Zhaohui Meng contributed equally to this work

*Corresponding authors. E-mail: aniruddhapatil135@gmail.com (A.P.), linnaibo@xmu.edu.cn (N.B.L.), phyliuxy@nus.edu.sg (X.Y.L.)

S1 Materials and Methods

S1.1 Chemical Reagents and Apparatus

In exception to wool fibers, all other chemicals are of analytical grade and used as received without further purification. Merino 64's wool fibers were bought from Tongxiang Dushi Woolen Material Co., Ltd. Chloroauric acid tetrahydrate ($HAuCl_4·4H_2O$, ≥47.8%), sodium bicarbonate ($NaHCO_3$, ≥99.5%), and ethanol (C_2H_6O, ≥99.7%) were from Sinopharm Chemical Reagent. Sodium dodecylsulfate (SDS, ≥99%), sodium sulfide nonahydrate ($Na_2S·9H_2O$, ≥98.0%), urea (CH_4N_2O, ≥99.0%), citric acid monohydrate ($C_6H_8O_7·H_2O$, ≥99.5%), disodium hydrogen phosphate dodecahydrate ($Na_2HPO_4.12H_2O$, ≥99.0%), sodium chloride (NaCl, ≥99.5%) and sodium hydroxide (NaOH, ≥96.0%) were obtained from Xilong Chemical Industry. Uric acid ($C_5H_4N_4O_3$, 99%) was obtained from Alfa Aesar. D-(+)-Glucose ($C_6H_{12}O_6$, batch no. C10100819, 99%), L-(+)- lactic acid ($C_3H_6O_3$, batch no. C10101039, 90%) and Poly(vinyl butyral) (PVB) (M.W90000-120000) was obtained from Shanghai Macklin Biochemical Co. Ltd. Nafion PFSA polymer (Dispersion D520 (5%)) was obtained from OU point Ltd. The water used in all experiments was ultrapure (the resistivity >18 MΩ cm^{-1}).

S1.2 Preparation of Regenerated WK Solution

The raw wool fibers were treated to extract WK via reduction method[1]. Accurately weighted 12.5 g pre-treated wool fibers were added in a 250 mL aqueous solution containing urea (4 M),

Na_2S (0.1 M), and SDS (0.02 M). The above mixture was stirred at 50 °C for 12 h with subsequent filtration. The filtrated stock was dialyzed against ultrapure water (18 MΩ) using a dialysis bag (Solarbio, molecular cut-off about 3500 Da) for 3 d. The dialyzed solution was concentrated at 60 °C for 8 h, and the concentration of WK solutions was 5 wt% on average. The obtained WK solutions were stored at 4 °C before further usage.

S1.3 Preparation of AuNCs@WK

In a typical experiment, 5 mL aqueous solution of $HAuCl_4$ (10×10^{-3} M) was added to 5 mL WK solution (2.5 wt%) under vigorous stirring. The pH of reaction mass is adjusted by addition of a 0.5 mL NaOH solution (1 M) 10-12, which was then retained at 45 °C for 12 h. The acquired AuNCs@WK were preserved at 4 °C for future application.

S1.4 Preparation of AuNCs@WK Composite Sponges

The composite mass is subjected to the freeze-drying method to sponge formation. The obtained suspension of AuNCs@WK (without dialysis) was transferred into a mold and frozen at −20 °C for 24 h. Followed to this the hybrid solidified mass was dried by a freeze-dryer (-106 °C) for 48 h to fabricate 3D porous composite sponges.

S1.5 Preparation of AuNPs@NPWC

The solid mass of AuNCs@WK is heated initially to 150 °C for 60 min in a tube furnace with N_2 atmosphere to take out water content, followed by heating to 350 °C (2 °C min^{-1}) for 60 min to develop the incipient conjugated carbon skeleton. It transformed into the polyaromatic N-doped porous carbon matrix with temperature rise from 350 to 700 °C at a rate of 2 °C min^{-1} and calcined for 2 h. The carbonized material was washed several times with DI water to remove salt residues. The black solid composite material was dried at 60 °C overnight.

S1.6 Characterization of AuNPs/NPWC

The crystallographic phase of the as-carbonized composite materials was characterized by X-ray diffraction (XRD, Bruker D8 AVANCE). The surface morphology, porosity and distribution of AuNPs in the composite materials were examined with a field emission gun scanning electron microscope (FEGSEM, Hitachi SU70) with an accelerating voltage of 5 kV. The carbon matrix, size and morphology of nanoparticles were characterized by transmission electron microscopy (TEM) through a JEM-2100F (JEOL, Japan). Nitrogen adsorption/desorption isotherms at 77.3 K were obtained using a Micromeritics TriStar II 3020 static volumetric analyzer. Prior to adsorption measurements, the samples were degassed for 12 h at 100 ºC ensuring that the residual pressure fell below 10 mbar. The Brunauer-Emmett-Teller surface area was calculated within the relative pressure range 0.05 to 0.2. Total volume was calculated at $P/P_0 = 0.99$. The surface element state and the constituent of N-functional groups were detected by X-ray photoelectron spectroscopy (Quantum 2000, PHI, USA), whereas, Raman spectra were taken using (HORIBA LabRAM HR Evolution) with a 532 nm excitation laser. Commercial handy UA meter of Sinocare made was used for UA comparative study, whereas pH comparison study has been performed by commercial pH meter of Thermo scientific made.

S2 Supplementary Tables and Figures

Table S1 Comparison of the elemental compositions of the carbonized materials carbonized at different carbonization temperature (C, H, N elemental analysis)

	C, H, N elemental analysis		
Material	C%	H%	N%
AuNPs@NPWC-500	47.89	1.602	9.281
AuNPs@NPWC-600	50.77	1.344	9.418
AuNPs@NPWC-700	51.95	2.055	8.385
AuNPs@NPWC-800	57.23	3.385	4.248

Table S2 Effect of carbonization temperature on S_{BET} area of the carbonized materials

Material	BET surface area $m^2 g^{-1}$
AuNPs@NPWC-400	27.0942
AuNPs@NPWC-500	381.2189
AuNPs@NPWC-600	452.6490
AuNPs@NPWC-700	625.0586
AuNPs@NPWC-800	447.6388

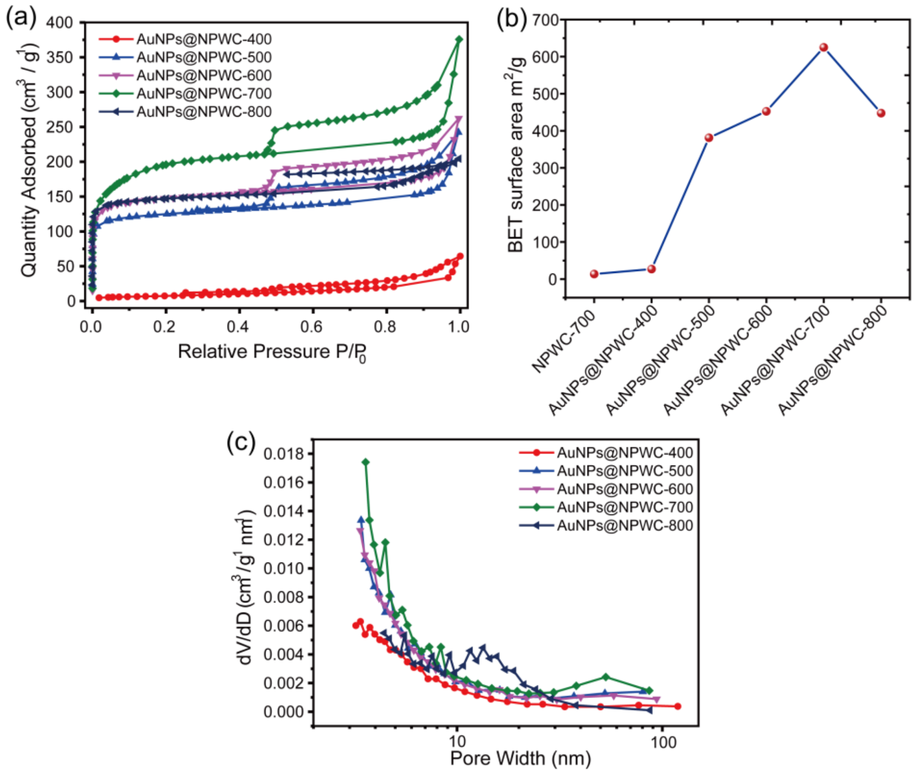

Fig. S1 (**a**) N_2 adsorption–desorption isotherms. (**b**) Effect of carbonization temperature on the BET surface area. (**c**) Pore size distributions

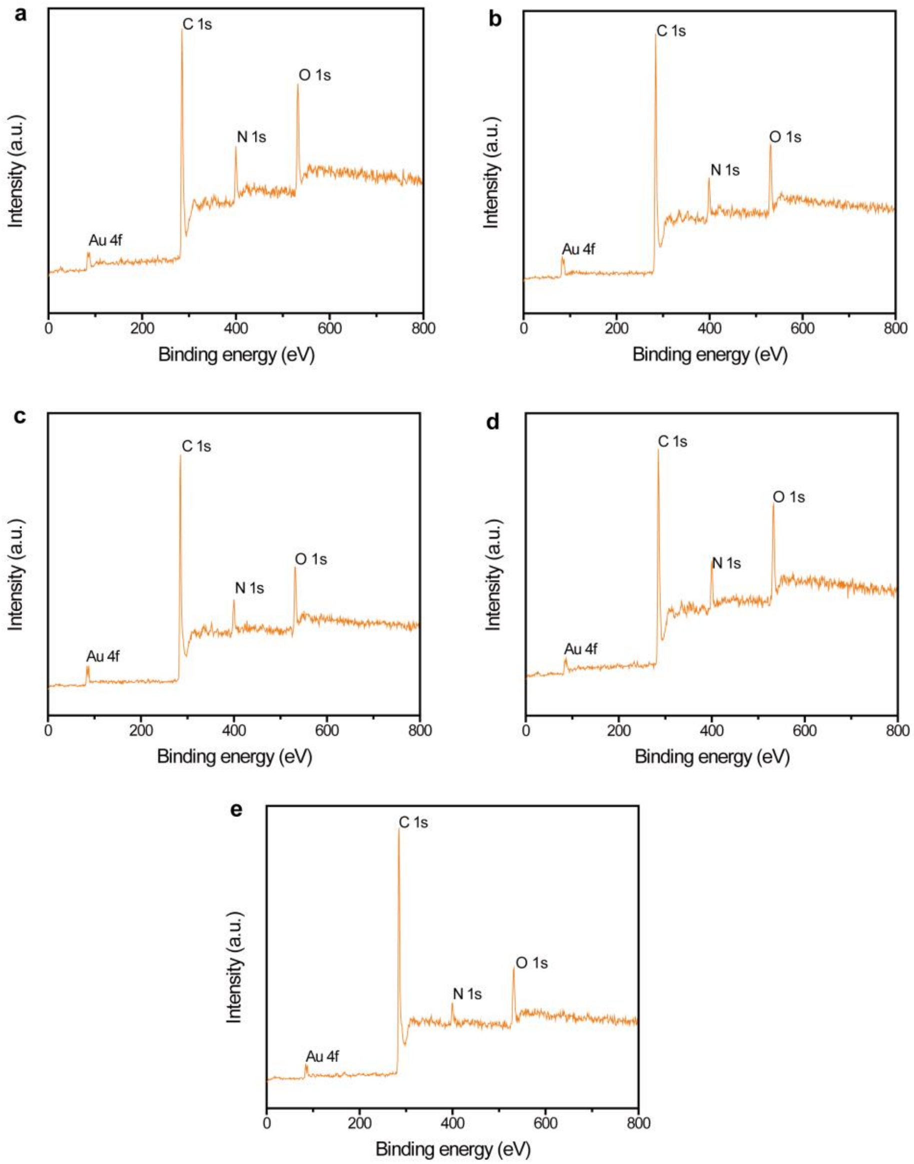

Fig. S2 Survey XPS data of (**a**) AuNPs@NPWC-400, (**b**) AuNPs@NPWC-500, (**c**) AuNPs@NPWC-600, (**d**) AuNPs@NPWC-700 and (**e**) AuNPs@NPWC-800

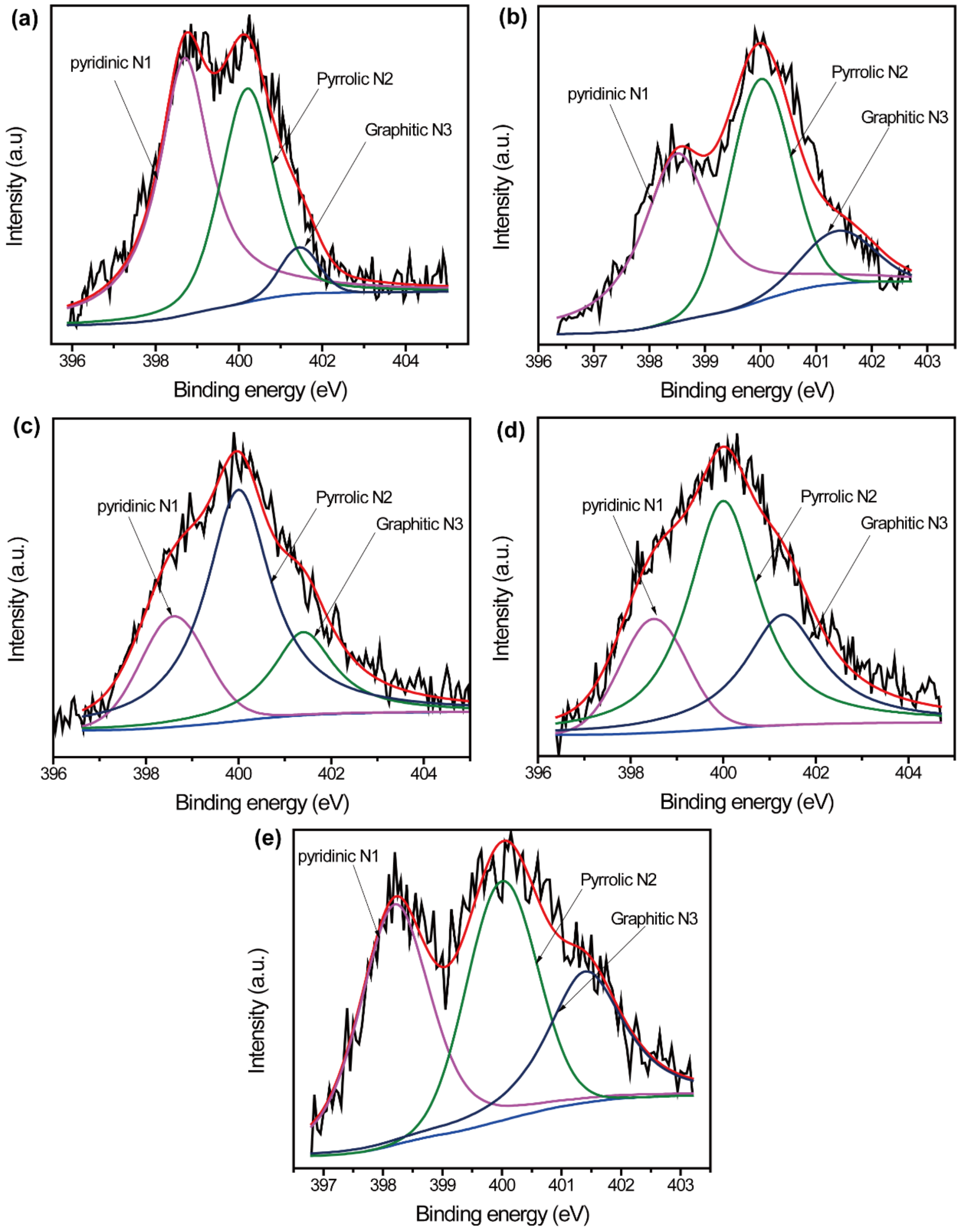

Fig. S3 High resolution N 1s XPS data of (**a**) AuNPs@NPWC-400, (**b**) AuNPs@NPWC-500, (**c**) AuNPs@NPWC-600, (**d**) AuNPs@NPWC-700 and (**e**) AuNPs@NPWC-800

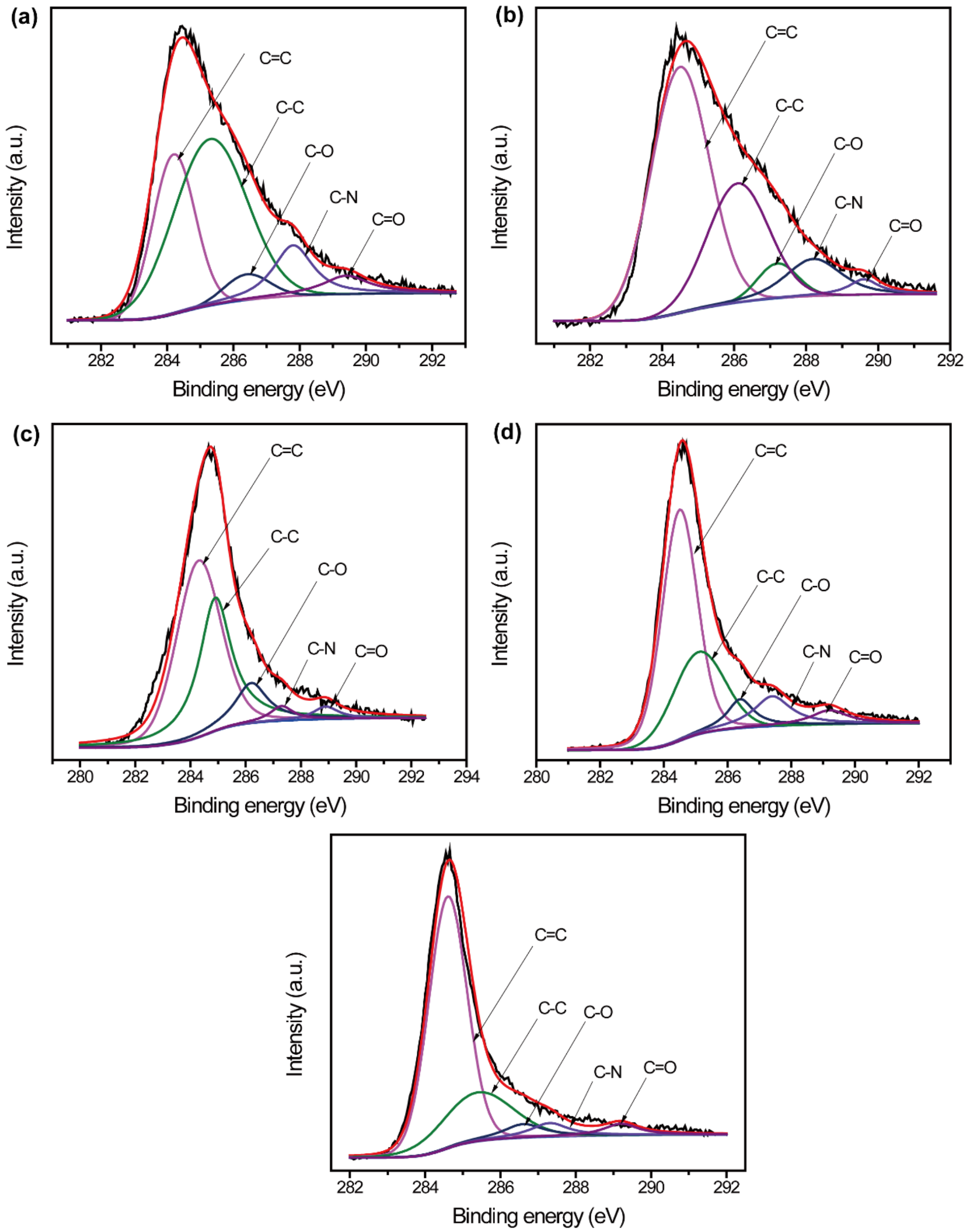

Fig. S4 High resolution C 1s XPS data of (**a**) AuNPs@NPWC-400, (**b**) AuNPs@NPWC-500, (**c**) AuNPs@NPWC-600, (**d**) AuNPs@NPWC-700 and (**e**) AuNPs@NPWC-800

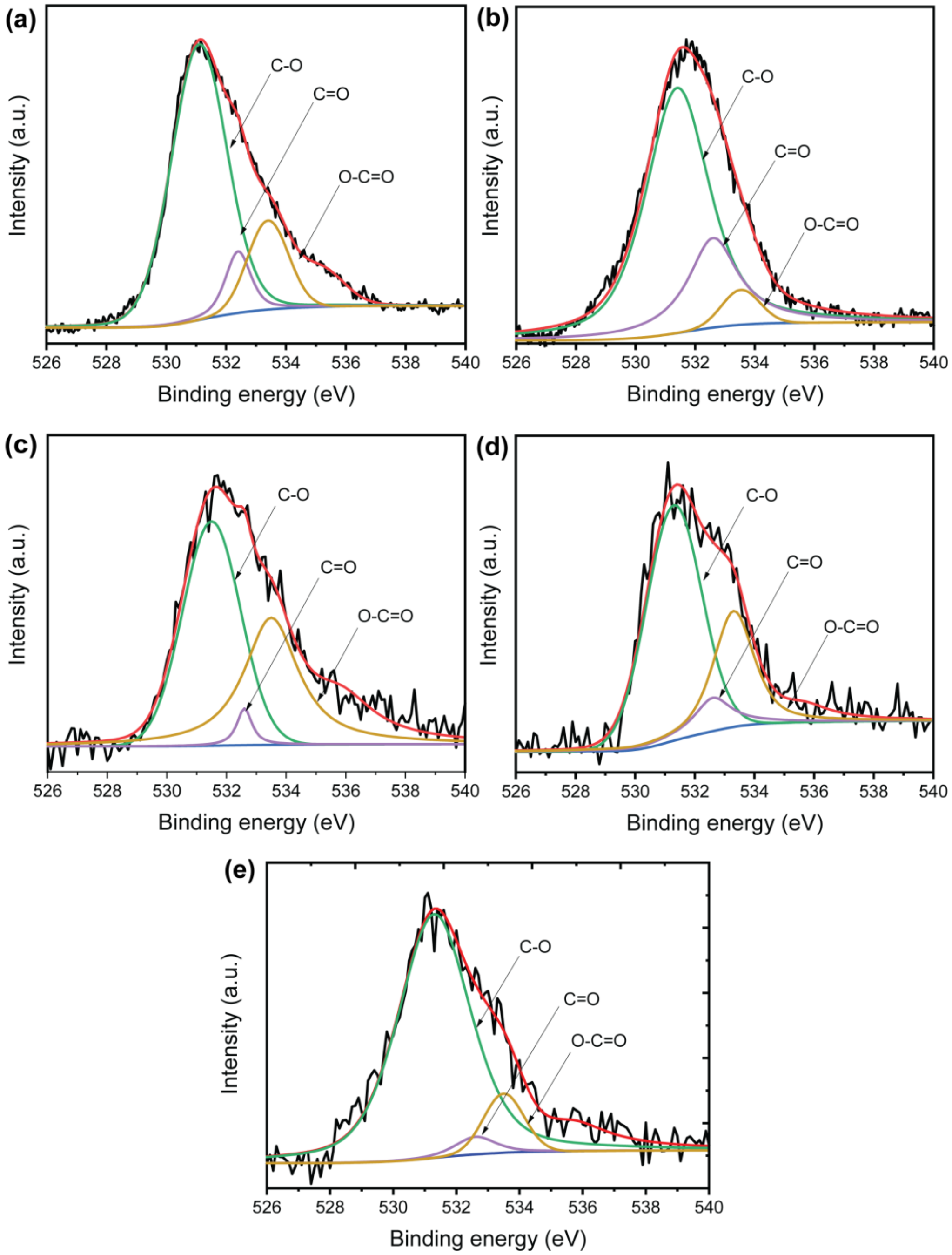

Fig. S5 High resolution O 1s XPS data of (**a**) AuNPs@NPWC-400, (**b**) AuNPs@NPWC-500, (**c**) AuNPs@NPWC-600, (**d**) AuNPs@NPWC-700 and (**e**) AuNPs@NPWC-800

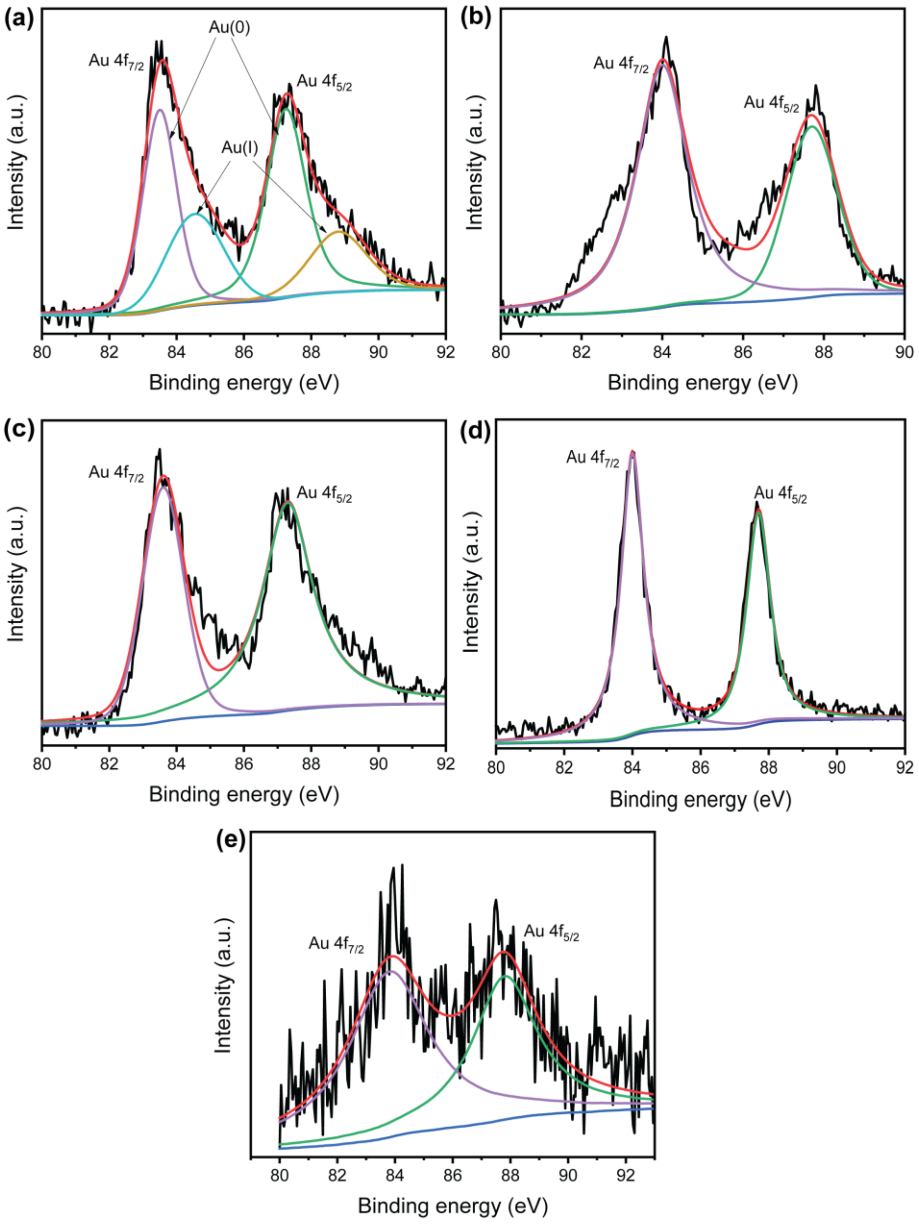

Fig. S6 XPS core level spectra of Au 4f of (**a**) AuNPs@NPWC-400, (**b**) AuNPs@NPWC-500, (**c**) AuNPs@NPWC-600, (**d**) AuNPs@NPWC-700 and (**e**) AuNPs@NPWC-800

Table S3 Comparison of the elemental compositions of the carbonized material based on XPS analysis

Material	Carbon %	Nitrogen %	Oxygen %	Au %
AuNPs@NPWC-400	76.7	9.53	12.97	0.8
AuNPs@NPWC-500	80.5	7.96	10.45	1.08
AuNPs@NPWC-600	82.33	7.63	9.14	0.9
AuNPs@NPWC-700	85.09	4.89	9.04	0.98
AuNPs@NPWC-800	86.21	4.27	9.17	0.34

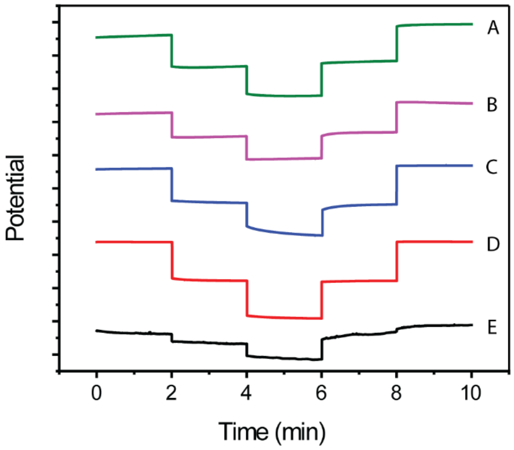

Fig. S7 Sensitivity comparison of carbonized composite materials (**A**) AuNPs@NPWC-800, (**B**) AuNPs@NPWC-700, (**C**) AuNPs@NPWC-600, (**D**) AuNPs@NPWC-500 and (**E**) AuNPs@NPWC-400

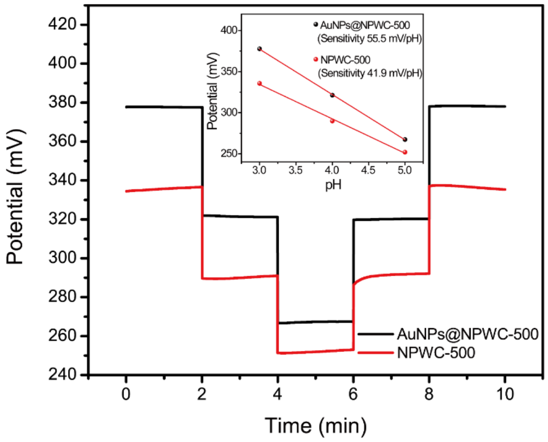

Fig. S8 Sensitivity comparison of AuNPs@NPWC-500 and NPWC-500 using McIlvaine's buffer pH 3 to 5

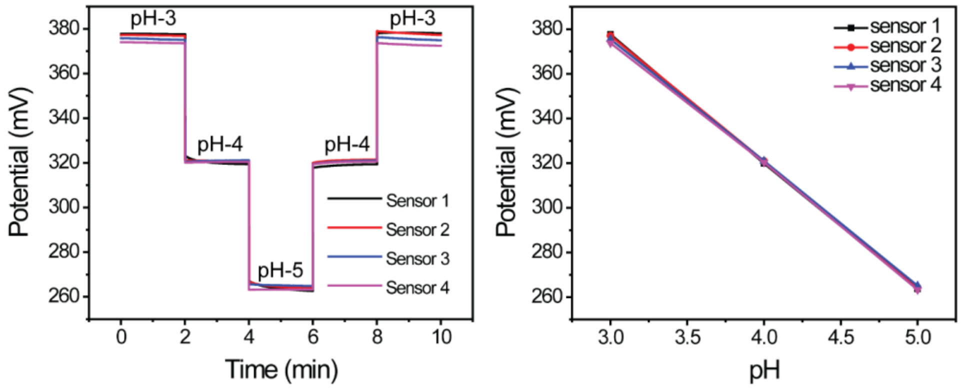

Fig. S9 Reproducibility study of AuNPs@NPWC-500 modified electrode for pH sensing

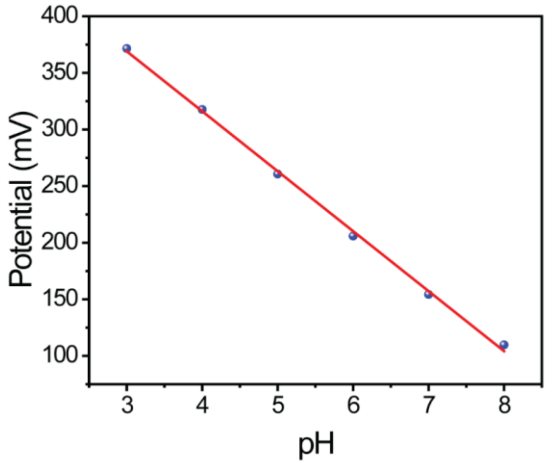

Fig. S10 Calibration curve showing pH Vs potential (mV) using AuNPs@NPWC-500 modified electrode (for identifications of real sample pH)

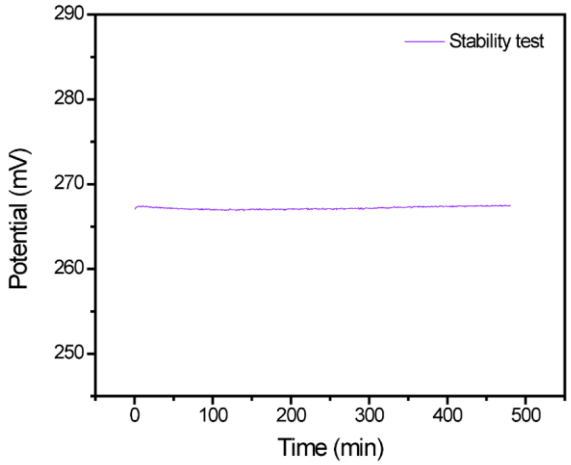

Fig. S11 Stability test of AuNPs@NPWC-500 electrode using McIlvaine's buffer pH 5

Table S4 Comparison of pH value detected by commercial pH meter and AuNPs@NPWC-500 electrode

Sample	pH sensor (This work)	pH meter
Sweat Sample 1	5.53	5.56
Sweat Sample 2	5.73	5.77
Sweat Sample 3	5.51	5.55
Sweat Sample 4	5.81	5.78
Sweat Sample 5	5.57	5.60
Urine Sample 1	5.65	5.68
Urine Sample 2	5.80	5.72
Urine Sample 3	5.70	5.69
Urine Sample 4	5.74	5.62
Urine Sample 5	5.49	5.51

Table S5 Comparison of different electrochemical sensor proposed for the determination of UA

Electrode material	Linear range (μM) UA	Detection limit (μM) UA	References
AuNPs-β-CD-Gra	0.5-60	0.21	S2
Au-RGO	8.8-53	1.8	S3
PdAg-NFs/RGO	1-150	0.081	S4
Fe_3O_4@Au-GA	1-300	0.05	S5
3DHA-AuNPs*	1-60	0.005	S6
RGO-ZnO	3-330	1.08	S7
AuNPs@NPWC-700	5-150	0.1	This work

*GA: graphene, RGO: reduced graphene oxide; NFs: nanofibers; 3DHA-AuNPs :3D-Graphene hydrogel – gold nanoparticles; RGO: reduced graphene oxide

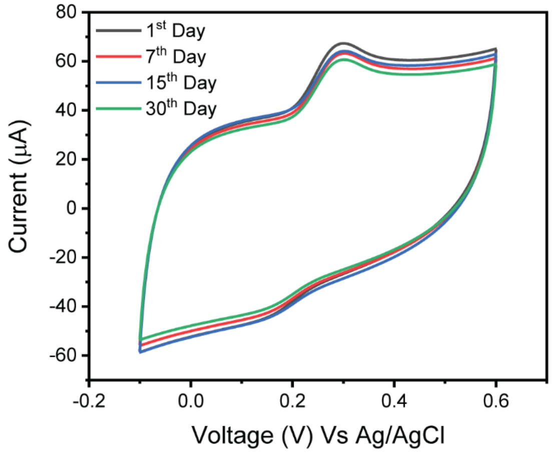

Fig. S12 Stability taste of AuNPs@NPWC-700 modified sensor for the determination of 1 mM UA

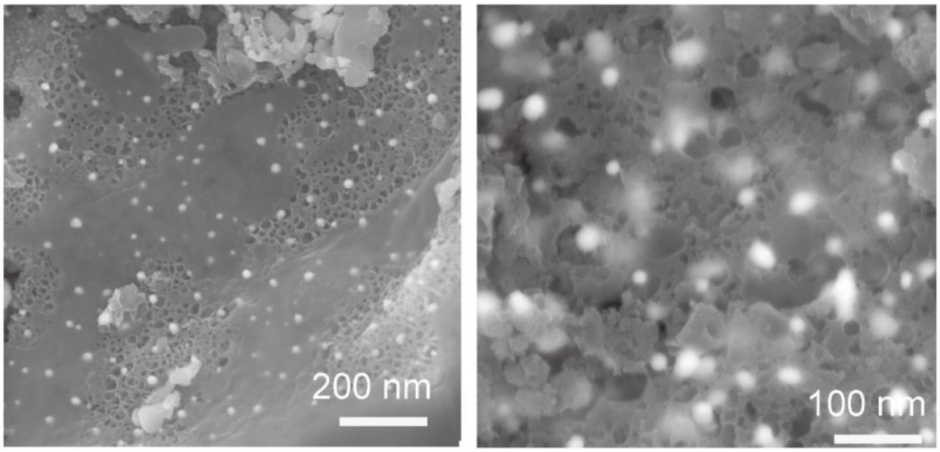

Fig. S13 SEM images of AuNPs@NPWC-700 catalyst before (left) and after (right) 100 cycles of CV in 0.1 M PBS (pH = 7.0) containing 0.1 mM UA

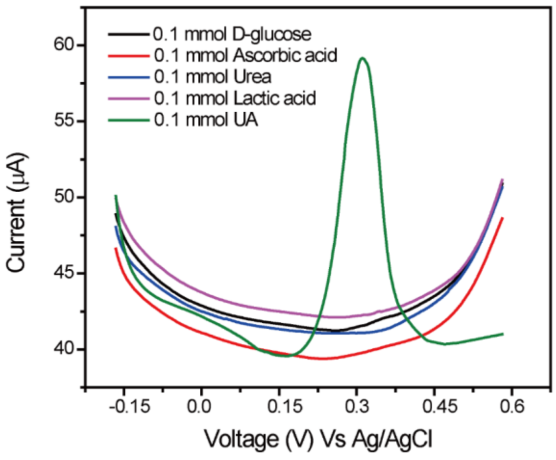

Fig. S14 Selectivity of AuNPs@NPWC-700 towards UA detection

Table S6 Comparison of UA value detected by commercial UA meter and AuNPs@NPWC-700 electrode

Sample	UA sensor (μM L^{-1})	Commercial UA meter (μM L^{-1})
Urine Sample 1	4240	4120
Urine Sample 2	3570	3530
Urine Sample 3	4678	4630
Urine Sample 4	5034	5140
Urine Sample 5	4787	4480
Urine Sample 6	4787	4800
Urine Sample 7	4128	4140
Urine Sample 8	4652	4440
Urine Sample 9	4082	3910
Urine Sample 10	3350	3250

Supplementary References

[S1] Y. Xing, C. Shi, J. Zhao, W. Qiu, N. Lin et al., Mesoscopic-functionalization of silk fibroin with gold nanoclusters mediated by keratin and bioinspired silk synapse. Small **13**, 1702390 (2017). https://doi.org/10.1002/smll.201702390

[S2] X.Q. Tian, C.M. Cheng, H.Y. Yuan, J. Du, D. Xiao, S.P. Xie, M.M.F. Choi, Simultaneous determination of L-ascorbic acid, dopamine and uric acid with gold nanoparticles-beta-cyclodextrin-graphene-modified electrode by square wave voltammetry. Talanta **93**, 79 (2012). https://doi.org/10.1016/j.talanta.2012.01.047

[S3] C.Q. Wang, J. Du, H.W. Wang, C.E. Zou, F.X. Jiang, P. Yang, Y.K. Du, A facile electrochemical sensor based on reduced graphene oxide and Au nanoplates modified glassy carbon electrode for simultaneous detection of ascorbic acid, dopamine and uric acid. Sens. Actuators B-Chem. **204**, 302 (2014). https://doi.org/10.1016/j.snb.2014.07.077

[S4] J.J. Jiang, X.Z. Du, Sensitive electrochemical sensors for simultaneous determination of ascorbic acid, dopamine, and uric acid based on Au@Pd-reduced graphene oxide nanocomposites. Nanoscale **6**, 11303 (2014). https://doi.org/10.1039/C4NR01774A

[S5] M.L. Liu, Q. Chen, C.L. Lai, Y.Y. Zhang, J.H. Deng, H.T. Li, S.Z. Yao, A double signal amplification platform for ultrasensitive and simultaneous detection of ascorbic acid, dopamine, uric acid and acetaminophen based on a nanocomposite of ferrocene thiolate stabilized Fe_3O_4@Au nanoparticles with graphene sheet. Biosens. Bioelectron. **48**, 75 (2013). https://doi.org/10.1016/j.bios.2013.03.070

[S6] Q. Zhu, J. Bao, D. Huo, M. Yang, C. Hou et al., 3D Graphene hydrogel – gold nanoparticles nanocomposite modified glassy carbon electrode for the simultaneous determination of ascorbic acid, dopamine and uric acid. Sens. Actuators B-Chem. **238**, 1316 (2017). https://doi.org/10.1016/j.snb.2016.09.116

[S7] X. Zhang, Y.C. Zhang, L.X. Ma, One-pot facile fabrication of graphene-zinc oxide composite and its enhanced sensitivity for simultaneous electrochemical detection of ascorbic acid, dopamine and uric acid. Sens. Actuator B-Chem. **227**, 488 (2016). https://doi.org/10.1016/j.snb.2015.12.073
